# *Cistanche deserticola* Addition Improves Growth, Digestibility, and Metabolism of Sheep Fed on Fresh Forage from Alfalfa/Tall Fescue Pasture

**DOI:** 10.3390/ani10040668

**Published:** 2020-04-12

**Authors:** Xulei Liu, Fuyao Liu, Tianhai Yan, Shenghua Chang, Metha Wanapat, Fujiang Hou

**Affiliations:** 1 State Key Laboratory of Grassland Agro-ecosystems, Key Laboratory of Grassland Livestock Industry Innovation, Ministry of Agriculture and Rural Affairs, College of Pastoral Agriculture Science and Technology, Lanzhou University, Lanzhou 730020, China; liuxl201906@163.com (X.L.); liuxl17@lzu.edu.cn (F.L.); cychangsh@lzu.edu.cn (S.C.); 2 Agri-Food and Biosciences Institute, Hillsborough, County Down BT26 6DR, UK; Tianhai.Yan@afbini.gov.uk; 3 Tropical Feed Resources Research and Development Center (TROFREC), Department of Animal Science, Faculty of Agriculture, Khon Kaen University, Khon Kaen 40002, Thailand; metha@kku.ac.th

**Keywords:** additives, NDF, body weight gain, dry matter intake, urine energy, enteric methane emission, grassland

## Abstract

**Simple Summary:**

*Cistanche deserticola* is a functional plant which mainly grows in desert and is parasitic on roots of the host species *Haloxylon ammodendron*. It has advantages in improving bodily intestinal peristalsis, immunity, anti-aging, anti-oxidation, and liver health and was supplied for sheep in this experiment to study the effects of *C. deserticola* addition on nutrients digestion, nitrogen balance, energy utilization, and methane production. The results revealed that *C. deserticola* has good utilization value in animal nutrition. The data are useful for further research on this natural plant additive to improve the health and productivity of the sheep fed on fresh forage from alfalfa/tall fescue pastures.

**Abstract:**

This study is targeted at evaluating whether *C. deserticola* addition promotes digestion, nitrogen and energy use, and methane production of sheep fed on fresh forage from alfalfa/tall fescue pastures. The sheep feeding trial was conducted with four addition levels with *C. deserticola* powder, and a basal diet of fresh alfalfa *(Medicago sativa)* and tall fescue (*Festuca arundinacea*). Addition levels of 4% and 6% improved average body weight gain (BWG) by 215.71 and 142.86 g/d, and feed conversion ratio (FCR) by 0.20 and 0.14, respectively. Digestibility of dry matter (DM), organic matter (OM), neutral detergent fiber (NDF), and ether extract (EE) was 62.25%, 65.18%, 58.75%, and 47.25% under the addition level of 2%, which is greater than that in the control group. *C. deserticola* addition improved energy utilization efficiency, while addition levels of 2% and 4% increased nitrogen intake and deposited nitrogen. Overall, *C. deserticola* has the potential to improve growth performance, digestion of sheep, so it has suitability to be used as a feed additive.

## 1. Introduction

The use of feed additives such as antibiotics, hormones, and chemicals to maximize ruminant animal performance [1], is common practice worldwide, with the aim of achieving better livestock health and cost-efficient livestock production [2]. However, this approach has become less socially acceptable recently, mostly due to the increasing risks at the quality and safety of animal food. Indeed such practices have now been restricted, while reducing antibiotics and searching for nutritive antibiotic alternatives have been deeply encouraged to focus on the use of native plants in animal agriculture in the European Union [3].

Functional plants as phytogenic additives in the ruminant feeds are a promising alternative to antibiotics [4]. Herbage grown in harsh environments, including desert environments, severe cold, and high altitudes, has abundant functional components related to secondary physiological metabolism. The use of functional plants as dietary addition is becoming more widely accepted in animal agriculture [5], as our understanding of their history and efficacy has increased. In particular, there is a long history and extensive knowledge of functional plants in certain parts of Asia. Functional herbs contain functional substances, enhance the disease resistance of livestock, and increase nutrient absorption, thus improving livestock growth and production [6]. Also, this strategy takes advantage of the low toxicity of herb-based supplements, the avoidance of drug resistance to antibiotics, and a reduction in active ingredient residue in livestock products such as meat and milk. Numerous studies have shown the beneficial effects of natural plant addition on feed intake, immune function, rumen fermentation, and productivity of dairy, beef cattle as well as in small ruminants. One of the studies indicated that 2% herbal additives (a mixture of Astragalus root, Angelica root, and Atractylodes rhizome) increased the body weight gain of sheep [7]. Some experiments have shown that the addition of *Fructus Ligustri Lucidi* at 300 or 500 mg/kg dry matter (DM) increased DM and organic matter (OM) digestibility of sheep [8]. Dietary addition of natural plant extracts can influence palatability, rumen microflora species, and population size of rumen microorganisms responsible for forage degradation [2]. Regarding environment and animal health, the effect of functional herbs on methane production during rumen fermentation has been interestingly evaluated. For example, the lateral branches of *Clerodendrum phlomidis* have the potential to decrease CH_4_ production with no side-effect on the ruminant health and production [9]. Saponins in some plant species were found to inhibit or suppress protozoa in the rumen and thus reduce ammonia and methane production [10].

In this study, *Cistanche deserticola*, one of the natural herbs, was chosen as a potential kind of herb additive to study its effects on nutrients intake, digestibility, and methane production of sheep. Many studies on *C. deserticola* were conducted in humans [11] and mice [12], but hardly in ruminants. *C. deserticola*, a desert plant unique to the arid regions in northwest China and Central Asia, parasitizes on roots of the host species *Haloxylon ammodendron* and is a functional plant that improves bodily intestinal peristalsis, immunity, anti-aging, anti-oxidation, and liver health [13]; no toxicity contributes to ruminant safety. The use of this plant will not cause ethical problems because *C. deserticola* is a common plant in this region, and it has been planted in a large area in the world. Saccharides occupy a high proportion in *C. deserticola*, among which polysaccharides, oligosaccharides, and galactitol are the main bioactive compounds [14], and there are also phenylethanoid glycoside, volatile components, iridoids, flavonoids, alkaloids in it. Galactitol is responsible for the laxative bioactivity [15] and may affect the nutrients in the dung of ruminants. The objective of this study is to provide a scientific basis for the development and utilization of natural plants addition in animal husbandry and methodological support for improving the digestion efficiency of sheep fed on fresh forage. As a complement to the existing studies regarding the medicinal benefits of *C. deserticola*, this study seeks to evaluate different inclusions of *C. deserticola* on nutrient digestion, energy balance, nitrogen balance, and methane production of sheep.

## 2. Materials and Methods

The animal sampling procedure strictly followed the rules and regulations of experimental field management protocols (file No: 2010-1 and 2010-2), which were approved by Lanzhou University. Sheep feeding trials were conducted at Linze Grassland Agriculture Station of Lanzhou University, located in the core area of the Heihe Oasis in Hexi Corridor, Northwest China (100°02′E, 39°15′N; 1390 m asl) [16]. The climate is a temperate continental climate, with distinct seasons, long cold winters, short hot summers, rapid warming in spring, and slow cooling in autumn. The annual average temperature is approximately 7.7 °C; annual average precipitation is 118.4 mm, over 70% concentrated from May to September; evaporation is 1830.4 mm. The dominant type of agricultural system is a specialized intensive cropping production system (SICP) and an extensively integrated crop–livestock production system (EICL). In this study, fresh forage of alfalfa and tall fescue was cut at the initial flowering period, and DM tested each morning at the Linze Research Station. *C. deserticola* was purchased from a herb company. The chemical composition of the fresh forage and *C. deserticola* are shown in Table 1.

### 2.1. Animals, Treatments, and Diets

Twenty-four 6-month-old rams with a mean BW of 27.51 ± 4.93 kg and good body condition were selected from a grazing flock at the start of the experiment period. Based on their initial body weight, the 24 sheep were allocated to each of the following four dietary treatments, and no significant difference among the average live weight of sheep existed in every treatment: (1) alfalfa-tall fescue, no addition (control, (CON), *n* = 6); (2) low level *C. deserticola* addition (2% DMI (DMI was determined pre-experimentally), CD 2%, *n* = 6); 3. medium level *C. deserticola* addition (4% DMI, CD 4%, *n* = 6), high-level *C. deserticola* addition (6% DMI, CD 6%, *n* = 6). Sheep in the CD 2%, CD 4%, and CD 6% groups were fed with the same basal diet of 60% alfalfa and 40% tall fescue (DM basis), which was 900 g in total, with low-level, mid-level, or high-level of *C. deserticola* addition, respectively. This experiment was conducted from July to August in 2018, including a 14-day pre-feeding period in penned groups and a 60-day experimental period in individual groups (including 42 days in the metabolic cages for digestion experiment and another 18 days in the respiration chambers for methane production experiment).

The DMI in each treatment was designed to supply maintenance and growth of 100 g/day live weight gain of male sheep according to tabular values listed in the CFSBC. Throughout this experimental period of 68 days, all sheep were housed in individual pens, given free access to water and salt licks, received natural light and ambient shade temperature. Coarsely chopped alfalfa and tall fescue fresh forage (5 to 10 cm length) were individually fed in the morning, noon, and night (07:00, 12:00 and 19:00), and *C. deserticola* powder was fed once a day (07:30) while feeding on fresh forage.

### 2.2. Respiration Chamber Description

Four direct open-circuit chambers were used with one sheep housed per chamber (LZUCKY-SDXCLZ-002, Institute of Grassland and Livestock Production System, Lanzhou University). Methane production, carbon dioxide production, and oxygen consumption for each were tested as the two-day average values for each sheep. The respiration chambers were made with plexiglass walls fitted in steel frames and mounted in a plastic leaky floor with two tubes for gas inlet and outlet, with a total volume of 4.86 m^3^ (1.98 m length, 1.46 m width, and 1.68 m height). Each individual chamber was equipped with a gas flow meter (GFM57, Aalborg, Orangeburg, New York, USA) to measure the flow rate, and the flow rates were set at a rate of 6 to 10 Nm^3^/h, which gave the concentrations of CO_2_, CH_4_, and O_2_ in the air samples within the appropriate measurement range recommended by the manufacturer. The concentration of CO_2_, CH_4_ and O_2_ for air from the atmosphere and exhaust gas leaving each chamber through a single port channel were determined by a gas analyzer (VA-3000, Horiba, Kyoto, Japan) on a rotational basis in 21 min internals. The gas was filtered through three filtrating apparatuses to ensure particles of number no more than 5 μm entered the gas analyzer. The analyzer was calibrated using standard gases (O_2_-free N_2_ and a known quantity of CO_2_, CH_4_, and O_2_, Dalian Special Gases Co., Ltd., Liaoning, China) at the start of the respiration measurement. The CO_2_, CH_4_, and O_2_ concentrations in air samples were determined in the absolute range of 0–2000 μL/L, 0–200 μL/L, and 0–25% (*v/v*), respectively. The recovery rates were in the range of 100 ± 2%. The production of CO_2_ and CH_4_ and the consumption of O_2_ were calculated by multiplying the flow rates by differences in the concentrations in the air samples before into and out of each individual chamber. The methane production was expressed as the average methane production (g/d) from 2-day measurements divided by metabolic body weight and dry matter intake.

### 2.3. Energy Balance

ME intake was calculated as the difference between GEI, excreted fecal energy (FE), and the sum of urinary energy (UE) and methane energy (CH_4_E) output. CH_4_ energy output per day was calculated by multiplying the volume of CH_4_ production per day by 0.03954 MJ/L. HP (kJ/day) was calculated with the following equation [17]:

HP (kJ/day) = 16.18 × O_2_ consumption (L/day) + 5.02 × CO_2_ production (L/day) − 2.17 × CH_4_ production (L/day) − 5.99 × N excretion (urinary N, g/day)

### 2.4. Sample Collection and Procedures

The body weight for each sheep was determined before the adaption period, before the sheep was moved in, and after the sheep was removed from the metabolism crate and chamber. Daily feed intake was measured by weighing both offered and residual forage daily throughout the experimental period. On day 15 of the experimental period, after the fourteen-day acclimation period for the feed we offered to the sheep, one sheep was randomly selected from each treatment group and moved to one of the four metabolic cages for seven days. On day 22, these sheep were moved to the individual groups in the shed, and another four sheep, randomly selected from the remaining sheep of the four treatment groups, entered the metabolic cages for digestion experiment. At the beginning and end of the period housed in metabolic cages, all sheep were weighed. Representative samples of alfalfa and tall fescue were collected at intervals throughout the digestion and metabolism experiment and composited for analysis of their feed quality indicator values expressed on a DM basis (Table 1). Digestion experiments were conducted on all 24 sheep for 6 days, following 1-day to adapt to the metabolism crate. During this period, total feces and urine were collected to determine daily urinary and fecal gross energy loss. Excreted urine (ca. 100 mL) was collected from each animal into a bottle with 50 mL of 10% (*v/v*) H_2_SO_4_ to maintain urine pH < pH 3, and stored at −20 °C in the refrigerator for further analysis. When all 24 sheep had finished 7 days of measurement, sheep were housed in a respiration chamber for methane production test. One sheep was randomly selected from each treatment group and moved to one of the four metabolic chambers for three days. Four indirect open-circuit respiration chambers were used with one sheep housed per chamber. CH_4_ production for each sheep was shown as the two-day average values for individual sheep. On day 4 in the period of metabolic chambers, these sheep were moved to the individual groups in the shed, and another four sheep randomly selected from the remaining sheep of the four treatment groups.

Rumen fluid samples were taken from each sheep 2 h post fresh forage and *C. deserticola* supply in the morning, using stomach tubing on the last day of feeding period. These collected samples were immediately measured for pH using a portable pH meter (PHBJ-260, Shanghai INESA Scientific Instrument Co., Ltd., Shanghai, China). Then the samples were strained through two layers of muslin and stored at −20 °C for volatile fatty acid (VFA) analysis. The VFA concentrations were determined by a gas chromatograph (Trace1300, Thermo Ltd., Rodano Milan, Italy) fitted with a polar capillary column.

### 2.5. Chemical Analysis

After the digestion experiment measurement, the stored feces samples of sheep were thawed at room temperature for 12 h, and the feces samples from each sheep over the six days were then mixed. A part of the thawed feces sample was used for the N measurement, according to the Association of Official Analytical Chemists method 976.05 [18]. CP concentration was calculated by multiplying nitrogen concentration by 6.25. The rest of the feces samples and collected fresh forage samples were dried in a forced ventilation oven at 65 °C for 48 h and then ground to pass through a 1-mm screen. A portion of each dried feces sample and mixed forage sample was used to measure ash by combustion using a muffle furnace at 550 °C for 5 h until all carbon was removed (method 942.05 [19]). Another part of each dried sample was finely ground to measure gross energy (GE), neutral detergent fiber (NDF), and acid detergent fiber (ADF). The GE was measured with an automatic calorimeter (6400, PARR Instrument Company, Moline, IL, USA). The NDF and ADF concentrations were analyzed sequentially in a fiber analyzer (ANKOM 2000, ANKOM Technology, Fairport, NY, USA) following the protocol described by Van Soest [19]. The urine samples from each sheep over the six days were also thawed at room temperature for 12 hours and then mixed before determining the urinary energy (UE) by using an automatic calorimeter (see above), and N was measured by using the Kjeldahl procedure described previously by the Association of Official Analytical Chemists [18]. For the UE measurement, 4 mL fully mixed urine was taken and absorbed by a filter paper of a known weight, and then the total energy of the filter paper with a urine sample was measured by an automatic calorimeter after it became dry at room temperature. There were another five samples using the same filter paper (known weight) to be measured for energy content, which was used to calculate the UE. The measurement of CP, NDF, ADF, and GE of the forage samples also followed the methods and instruments above. The ether extract of the forage samples was analyzed by using an extractor (ANKOM XT15, ANKOM Technology, Fairport, NY, USA).

### 2.6. Statistical Analysis

The effect of the treatment on the response variables was tested by one-way analysis of variance (ANOVA), and the means were separated using Tukey’s test at *p* = 0.05. Quadratic regression analysis in each part of the results was used to determine the relationship between the level of inclusion of *C. deserticola* and the response variables. The social science statistical software package version 20.0 (Chicago, Illinois, USA statistical software package company) was used to analyze the data.

## 3. Results

### 3.1. Feed Intake, Apparent Nutrient Digestibility and Body Weight Gain (BWG)

Average body weight gain (BWG) and feed conversion ratio (FCR) in the CD 4% and CD 6% groups were greater than those in the control group (*p* < 0.05). Intake of DM and OM per metabolic weight per day was greater in the CD 2% and CD 4% groups than that in the control group (*p* < 0.05). Intake of NDF per metabolic weight per day was greater in the CD 4% group than that in the control group (*p* < 0.05), but has no significant difference from that in the CD 2% and CD 6% groups. There was no significant effect on the intake of ADF and EE. The digestibility of DM, OM, and NDF was greater in the CD 2% group than that in the control group (*p* < 0.05), but has no significant difference from that in the CD 4% and CD 6% groups. EE digestibility was higher in the CD 2% group than that in the CD 4% group (*p* < 0.05). The addition of *C. deserticola* had no significant effect on the digestibility of ADF (*p* > 0.05; Table 2).

### 3.2. Energy Balance, Energy Utilization Efficiency and Methane Production

DE and ME were greater in the CD 2% and CD 4% groups than those in the control group (*p* < 0.05). *C. deserticola* addition had no significant effect on FE and UE (*p* > 0.05). The CH_4_E was greater in the CD 4% group than that in the control group (*p* < 0.05), but there was no significant difference between the CD 2% and CD 6% groups (*p* > 0.05). There was no significant difference in GE intake, HP, and RE between the control group and *C. deserticola* addition groups. GE digestibility and metabolic rates in the CD 2% group were greater than those in the control group (*p* < 0.05). The ratio of FE/GE intake was lower in the CD 2% group than that in the control group (*p* < 0.05), and the ratio of UE/GE intake was lower in the CD 2% and CD 4% groups than that in the control group (*p* < 0.05), but *C. deserticola* addition has no significant effect on the ratio of HP/GE intake and CH_4_E/ GE intake (Table 3).

### 3.3. Nitrogen Balance and Nitrogen Utilization Efficiency

Nitrogen intake and digestible nitrogen were greater in the CD 2% and CD 4% group than those in the control group (*p* < 0.05). There was no significant difference among the control, CD 2%, CD 4%, and CD 6% groups in fecal nitrogen, urine nitrogen, retained nitrogen, and the rates of retained nitrogen in total nitrogen intake (*p* > 0.05). There was a decrease in tendency in the ratio of FN/N intake and UN/N intake in the CD 2% and CD 4% groups (Table 4).

### 3.4. Rumen Fermentation Parameters

The rumen fluid pH was higher in the CD 4% and CD 6% groups than that in the control group (*p* < 0.05). The isobutyric acid, isovaleric acid, and valeric acid were higher in the CD 6% group than that in the control group (*p* < 0.05). There was no significant difference in total VFA, acetic acid, propionic acid, butyric acid, and the ratio of acetic acid/propionic acid between the control group and *C. deserticola* addition groups (Table 5).

### 3.5. Quadratic Models

Through quadratic equation fitting, the relationship between the level of inclusion of *C. deserticola* and response variables including intake, digestibility, energy parameters, and nitrogen parameters has certain regularity. The quadratic equations are shown in Table 6. The optimum inclusion level was calculated by the equation to identify the abscissa of the vertex in the quadratic equation, and is listed in Table 6 (*x* in the equation represents the inclusion of *C. deserticola*, and *y* in the equation represents response variables including intake, digestibility, energy parameters, and nitrogen parameters).

## 4. Discussion

### 4.1. Feed Intake, Digestibility, and BWG

Limited studies to date have examined the effects of *C. deserticola* addition on feed intake and nutrient digestibility in ruminants. Our study showed that DM intake and the digestibility of DM, OM, and NDF increased following the dietary addition of *C. deserticola*, and average body weight gain (BWG) and feed conversion ratio (FCR) in the CD 4% and CD 6% groups were greater than that in the control group. This could be explained by that chemical analysis of *C. deserticola* [20], which indicates that polysaccharides in the *C. deserticola* are one of the chemical components with properties most likely to influence sheep digestive physiology [21]. The results agree with the views in previous studies that polysaccharides in plants could affect ruminants’ feed intake and nutrient digestibility [22]. In a previous study, DM intake increased when lambs were fed 15 g/kg Astragalus polysaccharide [22]. Therefore, the increased DM intake and digestibility may be attributed to the effects of polysaccharides in *C. deserticola* on sheep. In addition, galactitol in *C. deserticola*, which has gentle laxative activity [14], has a function of improving bodily intestinal peristalsis, so the efficient intestinal peristalsis makes the digestion process of nutrients more effective and improves the absorption and utilization of nutrients. It is possible that higher feed conversion efficiency and nutrient digestibility will lead to an increase in sheep’s body weight.

### 4.2. Energy Balance

Plant additives develop their initial activity in the feed of ruminants as a flavor and can, therefore, influence eating patterns and gross energy intake [23]. Energy loss includes the form of urine, feces, and CH_4_ emissions in ruminants [24]. In our study, the ratio of FE output to GE intake was lower in the CD 2% group than that in the control group, and this could be explained by the improved DM digestibility because the less the DM excretion, the less the FE loss. The ratio of UE output to GE intake, which in previous studies was found to range from 0.9% to 4.8% [25], is an indispensable element of energy loss. UE output to GE intake was decreased in the CD 2% group. This showed that *C. deserticola* addition declined the loss of energy in urine, and, to some extent, the energy utilization efficiency was improved. CH_4_E was greater in the CD 4% group than in the control group, but there was no significant difference between CD 2%, CD 4%, and CD 6% groups. This could be explained by *C. deserticola* addition not affecting the methane production as a result of increasing feed intake, and there was no inhibitor of methane production in *C. deserticola*. The utilization efficiency of metabolism energy is attributed to metabolic capacity [26]; it may show that 2% *C. deserticola* addition improved the metabolic capacity of rumen, and, as a result, ME in the CD 2% group was higher than that in the control group. The positive effect on energy utilization is one of the important reasons why plant additives are widely applied in ruminants.

### 4.3. N Balance and N Utilization Efficiency

In our study, the N intake of sheep in the CD 2% and CD 4% groups was higher than that in the control group, and N egestion in feces had no significant difference between addition groups and control group, indicating higher N digestibility in the CD 2% and CD 4% groups compared with that in the control group. The results agree with the previous studies [27], which showed that increased CP intake resulted in enhanced N digestibility and elevated urinary N excretion in sheep [28], and this may be explained the hydrolysis of plant additives in rumen and reduced complex formation with protein, resulting in increased nitrogen through urine [29]. Retained nitrogen had an increasing tendency in *C. deserticola* addition group, so during the experimental period, sheep in all addition groups were in positive nitrogen balance; these results were the same as the studies on the impact of phytogenic feed additives on growth performance and nutrient digestion in growing livestock [30], but the results were different from the Zadbuke’s experiment, where he observed that there were no effects on nitrogen intake, nitrogen retention, and nitrogen balance by feeding a plant mixture in his study [31]. Reducing N output in urine is critical for reducing ammonia volatilization and N_2_O emissions, and thus improves the N efficiency for sustainable production. Most of the absorbable N supplied to the small intestine is provided by microbial protein synthesis in the rumen. Thus, the higher digestibility of N and higher excreted urinary N of sheep in the CD 2% group indicates a higher amount of synthetic microbial protein, suggesting that the bioactive components of *C. deserticola* play an important role in enhancing microbial activities in the rumen.

### 4.4. Methane Production and Ruminal Fermentation

Some compounds in plants such as condensed tannins [32], tea saponins [33], mulberry leaf flavonoids [34], were found to inhibit the rumen methanogenesis. On the contrary, methane production was not decreased with *C. deserticola* addition in our study. The possible reason was that methanogenesis inhibitors like condensed tannins, tea saponins, mulberry leaf flavonoids, and other potential inhibited components did not exist in *C. deserticola*. The 2% of DMI inclusion level of *C. deserticola* seemed to produce the least methane, whether per metabolic body weight or per dry matter intake; therefore, addition with 2% of DMI *C. deserticola* can reduce methane production as much as possible.

Ruminal pH is an important indicator of the rumen microbial ecosystem. Lower ruminal pH is a limiting factor to the establishment of a balanced microbial population and has a negative effect on fiber digestion via reduced microbial attachment [35]. Our results showed that *C. deserticola* addition increased ruminal fluid pH on a small scale, but that values are in the optimal range for rumen fermentation.

VFAs supply much of the energy needs of ruminants. Polysaccharides in plants are important energy and carbon sources of rumen microbes [36]. The polysaccharides in Astragalus cicer inhibited ruminal cellulose fermentation and depressed fiber utilization [37], and total VFA concentrations were affected by an interaction effect between the dietary treatment of different Astragalus polysaccharide supplementation and feeding time [22]. On the contrary, total VFA concentrations were not influenced by *C. deserticola* addition, although polysaccharides are one of the bioactive components in *C. deserticola*. Moreover, *C. deserticola* addition did not change rumen fermentation patterns to favor propionate, and not increase propionate concentrations although had a reduction tendency in the acetate to propionate rate in rumen fluid, so apparent digestibility of NDF was not decreased.

### 4.5. Quadratic Models

We wanted to calculate the optimum *C. deserticola* addition level (% DMI) according to the data in our study through the quadratic equation fitting. It is found that the intake of DM and OM, the digestibility of DM, OM and NDF, some energy parameters, and nitrogen parameters have certain regularity, the curve showed a parabolic distribution with downward opening and the best peak value, so the addition level of *C. deserticola* has an optimum value. The optimum inclusion level was calculated by the equation to identify the abscissa of the vertex in the quadratic equation, and according to the results we calculated, the optimum inclusion level of *C. deserticola* may be about 30 g/d (3.3% of DMI) on average, but further experiments should be done to identify the addition level more precisely in the future. If we use the optimum addition level in ruminants, we could find the highest potential to suit in their growth performance and digestion.

## 5. Conclusions

Results from this study show that addition diets of male sheep with *C. deserticola* at 2% and 4% of DMI addition level resulted in improved nutrient intake and apparent nutrient digestibility, also improved digestive nitrogen, digestive energy, and metabolism energy, suggesting that, under the experimental conditions of this study, *C. deserticola* has advantages to improve feed conversion efficiency, with no negative side-effect on rumen health. The optimum inclusion level was calculated by the quadratic equations between the inclusion of *C. deserticola* and response variables was about 30 g/d (3.3% of DMI addition level) on average. However, the addition of *C. deserticola* did not decrease the methane production of sheep. Further research and long-term studies are needed to validate the dietary effects of *C. deserticola*, and to confirm whether its bioactive components are transferred to the animal food products such as milk and meat. Following the trend of developing and utilizing new healthy natural functional plants, rationally utilizing *C. deserticola* is likely to be an effective way to improve the dietary efficiency of sheep fed on fresh forage.

## Figures and Tables

**Table 1:** Chemical composition of feed ingredients of experimental diets (dry matter (DM) basis).

Item	Chemical Composition	Mean Value
Fresh forage mixed with alfalfa and tall fescue	Dry matter (%)	52.45
	Organic matter (%)	89.41
	Crude protein (%)	12.94
	Neutral detergent fiber (%)	55.03
	Acid detergent fiber (%)	33.73
	Ether extract (%)	2.09
	Gross energy (MJ·kg^−1^)	16.82
*Cistanche* *deserticola*	Dry matter (%)	90.31
	Organic matter (%)	82.25
	Crude protein (%)	15.77
	Neutral detergent fiber (%)	28.40
	Acid detergent fiber (%)	16.75
	Ether extract (%)	0.33
	Gross energy (MJ·kg^−1^)	14.73

**Table 2:** Effect of *C. deserticola* addition on growth performance, intake, and digestibility (mean ± SE).

Items	*C. deserticola* Addition Level (% DMI)			
	0	2	4	6
**Growth Performance**				
BWG (g·d^−1^)	−79.04 ± 71.81 ^b^	66.67 ± 68.00 ^ab^	215.71 ± 30.66 ^a^	142.86 ± 94.20 ^a^
FCR (g BWG/g DMI)	−0.10 ± 0.08 ^b^	0.06 ± 0.07 ^ab^	0.20 ± 0.02 ^a^	0.14 ± 0.09 ^a^
**Intake (g/kg BW^0.75^/d)**				
DM	74.74 ± 3.05 ^b^	84.65 ± 3.71 ^a^	85.22 ± 3.76 ^a^	78.48 ± 1.90 ^ab^
OM	67.03 ± 2.67 ^b^	75.88 ± 3.49 ^a^	76.39 ± 3.56 ^a^	70.39 ± 1.72 ^ab^
NDF	40.81 ± 2.49 ^b^	46.34 ± 1.86 ^ab^	46.68 ± 1.98 ^a^	42.95 ± 1.31 ^ab^
ADF	25.03 ± 1.65	28.37 ± 1.33	28.58 ± 1.33	26.06 ± 0.87
EE	1.57 ± 0.14	1.78 ± 0.11	1.79 ± 0.12	1.65 ± 0.08
**Digestibility (%)**				
DM	56.48 ± 1.74 ^b^	62.25 ± 2.12 ^a^	61.84 ± 2.11 ^ab^	60.23 ± 1.81 ^ab^
OM	58.88 ± 1.71 ^b^	65.18 ± 2.04 ^a^	64.50 ± 2.00 ^ab^	62.68 ± 1.68 ^ab^
NDF	51.23 ± 1.53 ^b^	58.75 ± 2.83 ^a^	57.49 ± 3.02 ^ab^	56.27 ± 2.44 ^ab^
ADF	53.80 ± 2.74	55.38 ± 2.86	53.96 ± 3.02	50.33 ± 2.44
EE	39.47 ± 2.79 ^ab^	47.25 ± 3.51 ^a^	34.00 ± 4.57 ^ab^	33.38 ± 3.65 ^b^

Note: BWG, body weight gain; FCR, feed conversion ratio (ratio of BWG divided by the total DMI); a, b, and c mean within the same row with the different letters are significantly different (*p* < 0.05). DM, dry matter; OM, organic matter; NDF, neutral detergent fiber; ADF, acid detergent fiber; EE, ether extract.

**Table 3:** Effect of *C. deserticola* addition on methane production, energy balance, and energy utilization efficiency (mean ± SE).

Items	*C. deserticola* Addition Level (% DMI)			
	0	2	4	6
**CH_4_ Production**				
CH_4_ Production (g/kg BW^0.75^/d)	0.91 ± 0.06 ^b^	1.06 ± 0.05 ^ab^	1.18 ± 0.05 ^a^	1.05 ± 0.07 ^ab^
CH_4_ Production (g/kg DMI/d)	11.98 ± 0.87	12.02 ± 0.89	13.23 ± 0.52	13.27 ± 1.21
**Energy Balance**				
GE intake (MJ/kg BW^0.75^/d)	1.26 ± 0.09	1.43 ± 0.07	1.44 ± 0.07	1.33 ± 0.03
FE output (MJ/kg BW^0.75^/d)	0.53 ± 0.03	0.51 ± 0.03	0.53 ± 0.03	0.51 ± 0.02
UE output (MJ/kg BW^0.75^/d)	0.03 ± 0.00	0.02 ± 0.00	0.02 ± 0.00	0.03 ± 0.00
CH_4_E (MJ/kg BW^0.75^/d)	0.04 ± 0.00 ^b^	0.05 ± 0.00 ^ab^	0.06 ± 0.00 ^a^	0.05 ± 0.00 ^ab^
DE (MJ/kg BW^0.75^/d)	0.68 ± 0.07 ^b^	0.87 ± 0.06 ^a^	0.85 ± 0.06 ^a^	0.77 ± 0.03 ^ab^
ME intake (MJ/kg BW^0.75^/d)	0.66 ± 0.06 ^b^	0.84 ± 0.06 ^a^	0.83 ± 0.06 ^a^	0.74 ± 0.03 ^ab^
HP (MJ/kg BW^0.75^/d)	0.59 ± 0.08	0.61 ± 0.09	0.67 ± 0.12	0.63 ± 0.13
RE (MJ/kg BW^0.75^/d)	0.07 ± 0.13	0.23 ± 0.11	0.17 ± 0.15	0.11 ± 0.13
**Energy Utilization Efficiency**				
DE/GE intake (MJ/MJ)	53.88 ± 2.06 ^b^	60.43 ± 2.13 ^a^	59.14 ± 2.03 ^ab^	57.68 ± 1.75 ^ab^
ME/GE intake (MJ/MJ)	51.70 ± 2.09 ^b^	58.83 ± 2.22 ^a^	57.40 ± 2.07 ^ab^	55.64 ± 1.73 ^ab^
FE/GE intake (MJ/MJ)	42.47 ± 1.84 ^a^	35.89 ± 2.11 ^b^	36.82 ± 2.05 ^ab^	38.41 ± 1.63 ^ab^
UE/GE intake (MJ/MJ)	2.18 ± 0.14 ^a^	1.61 ± 0.13 ^c^	1.75 ± 0.04 ^bc^	2.03 ± 0.15 ^ab^
HP/GE intake (MJ/MJ)	49.98 ± 10.40	43.68 ± 7.21	47.20 ± 9.94	47.71 ± 9.55
CH_4_E/GE intake (MJ/MJ)	3.64 ± 0.27	3.66 ± 0.28	4.03 ± 0.16	3.91 ± 0.29

Note: GE, gross energy; ME, metabolizable energy; FE, fecal energy; UE, urinary energy; CH4-E, methane energy; HP, heat production; RE, retained energy. a, b, and c mean within the same row with the different letters are significantly different (*p* < 0.05).

**Table 4:** Effect of *C. deserticola* addition on nitrogen balance and nitrogen utilization efficiency (mean ± SE).

Items	*C. deserticola* Addition Level (% DMI)			
	0	2	4	6
**Nitrogen Balance**				
N intake (g/kg BW^0.75^/d)	1.57 ± 0.10 ^b^	1.81 ± 0.07 ^a^	1.82 ± 0.08 ^a^	1.68 ± 0.04 ^ab^
FN (g/kg BW^0.75^/d)	0.44 ± 0.03	0.45 ± 0.02	0.44 ± 0.01	0.44 ± 0.01
UN (g/kg BW^0.75^/d)	0.44 ± 0.11	0.48 ± 0.05	0.50 ± 0.10	0.54 ± 0.11
DN (g/kg BW^0.75^/d)	1.12 ± 0.09 ^b^	1.36 ± 0.08 ^a^	1.38 ± 0.09 ^a^	1.24 ± 0.04 ^ab^
RN (g/kg BW^0.75^/d)	0.68 ± 0.11	0.87 ± 0.08	0.88 ± 0.11	0.70 ± 0.11
**Nitrogen Utilization Efficiency**				
DN/N intake (%)	71.45 ± 1.85	74.82 ± 1.34	75.33 ± 1.50	73.88 ± 0.80
FN/N intake (%)	28.55 ± 1.85	25.18 ± 1.34	24.67 ± 1.50	26.11 ± 0.80
UN/N intake (%)	28.42 ± 6.04	26.79 ± 2.81	27.11 ± 5.06	32.11 ± 6.41
Retained N/N intake (%)	43.03 ± 5.48	48.03 ± 3.36	48.21 ± 4.83	41.78 ± 6.41

Note: N intake, nitrogen intake; FN, fecal N; UN, urinary N; RN, retained N. a, b, and c mean within the same row with the different letters are significantly different (*p* < 0.05).

**Table 5:** Effect of *C. deserticola* addition on rumen fermentation parameters (mean ± SE).

Items	*C. deserticola* Addition Level (% DMI)			
	0	2	4	6
pH	6.06 ± 0.01 ^c^	6.15 ± 0.01 ^b^	6.23 ± 0.01 ^a^	6.21 ± 0.02 ^a^
Total VFA (mmol·L^−1^)	81.30 ± 10.41	81.64 ± 12.02	81.53 ± 4.78	68.11 ± 18.76
Acetic acid (%)	73.66 ± 0.66	71.88 ± 0.74	72.71 ± 0.95	71.74 ± 0.46
Propionic acid (%)	17.46 ± 0.64	18.25 ± 0.46	17.83 ± 0.66	17.32 ± 0.44
Isobutyric acid (%)	1.66 ± 0.16 ^b^	1.84 ± 0.14 ^ab^	1.85 ± 0.16 ^ab^	2.27 ± 0.25 ^a^
Butyric acid (%)	4.98 ± 0.59	5.45 ± 0.56	5.15 ± 0.64	5.60 ± 0.39
Isovaleric acid (%)	1.61 ± 0.15 ^b^	1.90 ± 0.18 ^ab^	1.80 ± 0.11 ^ab^	2.30 ± 0.28 ^a^
Valeric acid (%)	0.64 ± 0.04 ^b^	0.69 ± 0.03 ^ab^	0.67 ± 0.05 ^ab^	0.77 ± 0.04 ^a^
Acetic acid/Propionic acid	4.25 ± 0.17	3.96 ± 0.13	4.11 ± 0.19	4.16 ± 0.11

Note: VFA, volatile fatty acid. a, b, and c mean within the same row with the different letters are significantly different (*p* < 0.05).

**Table 6:** Quadratic models between inclusion of *C. deserticola* and response variables.

Items	Equation	The Optimum Inclusion Level	*p*-Value	*R* ^2^
**Intake (g/kg BW^0.75^/d)**				
DM	=−0.013 x^2^ + 0.760 x + 74.836	29.23	0.043	0.136
NDF	=−0.007 x^2^ + 0.423 x + 40.868	30.21	0.049	0.164
**Digestibility (%)**				
DM	=−0.006 x^2^ + 0.368 x + 56.729	30.67	0.045	0.125
OM	=−0.006 x^2^ + 0.398 x + 59.167	33.17	0.047	0.166
NDF	=−0.007 x^2^ + 0.441 x + 51.666	31.50	0.035	0.134
**Energy Parameters (MJ/kg BW^0.75^/d)**				
DE	=−0.0002 x^2^ + 0.013 x + 0.691	32.50	0.043	0.173
ME	=−0.0002 x^2^ + 0.013 x + 0.664	32.50	0.046	0.184
**Nitrogen Parameters (g/kg BW^0.75^/d)**				
N intake	=−0.0003x^2^ + 0.018 x + 1.570	30.00	0.045	0.170
DN	=−0.0003 x^2^ + 0.017 x + 1.126	28.33	0.044	0.186

Note: DM, dry matter; NDF, neutral detergent fiber; OM, organic matter; DE, digestible energy; ME, metabolic energy; CH_4_E, methane energy; N intake, nitrogen intake; DN, digestible nitrogen. The “x” in the equation represents inclusion of *C. deserticola*. Unit of the optimum inclusion level is g/d.
